# Supplementary material for: CCR5 promotes the migration of pathological CD8+ T cells to the leishmanial lesions
Source: PLoS Pathog. 2024 May 6;20(5):e1012211. doi: 10.1371/journal.ppat.1012211 (PMC11098486; doi:10.1371/journal.ppat.1012211)
Supplement: S1 Fig — At day 90 after the start of treatment, patients with complete re-epithelialization of lesions and the resolution of inflamed borders were considered cured, or patients with active lesions at 90 days were defined as failing, <90d or >90d, respectively. Data was obtained from RNA-Seq analysis of lesions from 21 patients and 7 healthy skin. Gene expression is represented as counts per million (CPM) in the log2 scale. Statistical significance was determined using two-tailed unpaired Student’s t-test. *p < 0.05. (DOCX) [file ppat.1012211.s001.docx]

**S1 Fig. *CCL3* and *CCL4* expression at the lesions of patients that cured versus failed the pentavalent antimony treatment.** At day 90 after the start of treatment, patients with complete re-epithelialization of lesions and the resolution of inflamed borders were considered cured, or patients with active lesions at 90 days were defined as failing, <90d or >90d, respectively. Data was obtained from RNA-Seq analysis of lesions from 21 patients and 7 healthy skin. Gene expression is represented as counts per million (CPM) in the log2 scale. Statistical significance was determined using two-tailed unpaired Student's t-test. **p < 0.05.*
