# Supplementary material for: CCR5 promotes the migration of pathological CD8+ T cells to the leishmanial lesions
Source: PLoS Pathog. 2024 May 6;20(5):e1012211. doi: 10.1371/journal.ppat.1012211 (PMC11098486; doi:10.1371/journal.ppat.1012211)
Supplement: S2 Fig — Correlation between CCR5 expression and GZMB, GZMA, GZMH, PRF1, and GNLY expression between CL patients (A) and healthy skin (B). Data was obtained from RNASeq analysis of lesions from 21 patients and 7 healthy skin. Gene expression is represented as counts per million (CPM) in the log2 scale. Pearson correlation coefficient was used to determine the correlation between log2 expressions of CCR5 from human skin. *p < 0.05, **p ≤ 0.01, ***p ≤ 0.001, ****p < .0001. (DOCX) [file ppat.1012211.s002.docx]

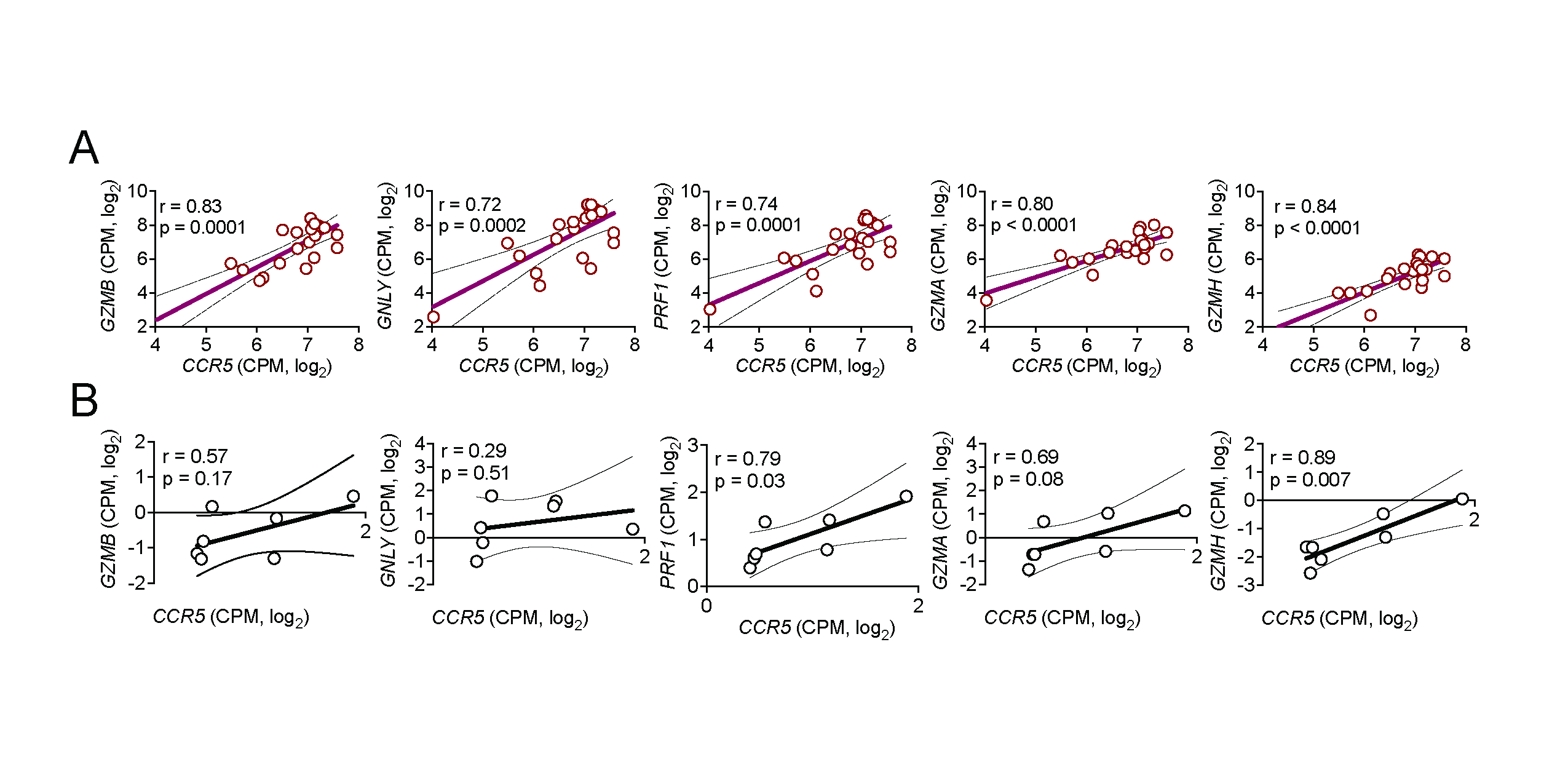


**S2 Fig. Correlations between *CCR5* expression and cytolytic molecules in CL patients and healthy skin.** Correlation between *CCR5* expression and *GZMB*, *GZMA*, *GZMH*, *PRF1*, and *GNLY* expression between CL patients (A) and healthy skin (B). Data was obtained from RNASeq analysis of lesions from 21 patients and 7 healthy skin. Gene expression is represented as counts per million (CPM) in the log2 scale. Pearson correlation coefficient was used to determine the correlation between log2 expressions of *CCR5* from human skin. **p < 0.05, **p ≤ 0.01, ***p ≤ 0.001, ****p < .0001.*
