# Supplementary material for: CCR5 promotes the migration of pathological CD8+ T cells to the leishmanial lesions
Source: PLoS Pathog. 2024 May 6;20(5):e1012211. doi: 10.1371/journal.ppat.1012211 (PMC11098486; doi:10.1371/journal.ppat.1012211)
Supplement: S3 Fig — Correlation between CCR5 expression and MCP counter abundance for T cells, monocytes/macrophages, neutrophils, and mDC at the L. braziliensis-lesion. Data was obtained from RNA-seq analysis of lesions from 21 patients. Gene expression is represented as counts per million (CPM) in the log2 scale. Pearson correlation coefficient was used to determine the correlation between log2 expressions of CCR5 from human skin. *p < 0.05, **p ≤ 0.01, ***p ≤ 0.001, ****p < .0001. (DOCX) [file ppat.1012211.s003.docx]

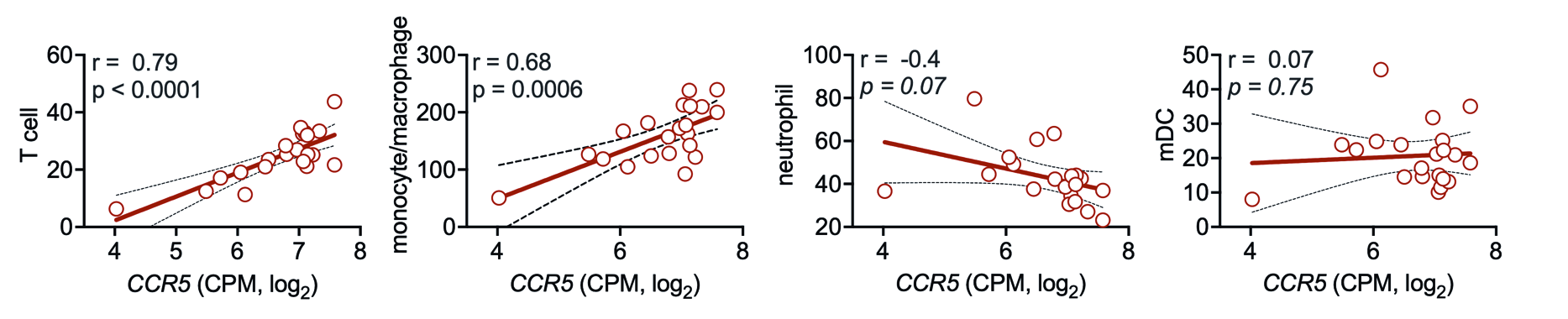


**S3 Fig. Correlations between CCR5 expression and estimated cell abundances at the *L. braziliensis*-lesions.** Correlation between *CCR5* expression and MCP counter abundance for T cells, monocytes/macrophages, neutrophils, and mDC at the *L. braziliensis*-lesion. Data was obtained from RNASeq analysis of lesions from 21 patients. Gene expression is represented as counts per million (CPM) in the log2 scale. Pearson correlation coefficient was used to determine the correlation between log2 expressions of *CCR5* from human skin. **p < 0.05, **p ≤ 0.01, ***p ≤ 0.001, ****p < .0001.*
