# Supplementary material for: CCR5 promotes the migration of pathological CD8+ T cells to the leishmanial lesions
Source: PLoS Pathog. 2024 May 6;20(5):e1012211. doi: 10.1371/journal.ppat.1012211 (PMC11098486; doi:10.1371/journal.ppat.1012211)
Supplement: S4 Fig — Representative dot plots and graph bars of CD11b+ cells expressing CCR5 in the lesion and dLN of L. braziliensis-infected Rag1-/- reconstituted with CD8+ T cells at 5 weeks post-infection. Statistical significance was determined using two-tailed unpaired Student’s t-test. (DOCX) [file ppat.1012211.s004.docx]

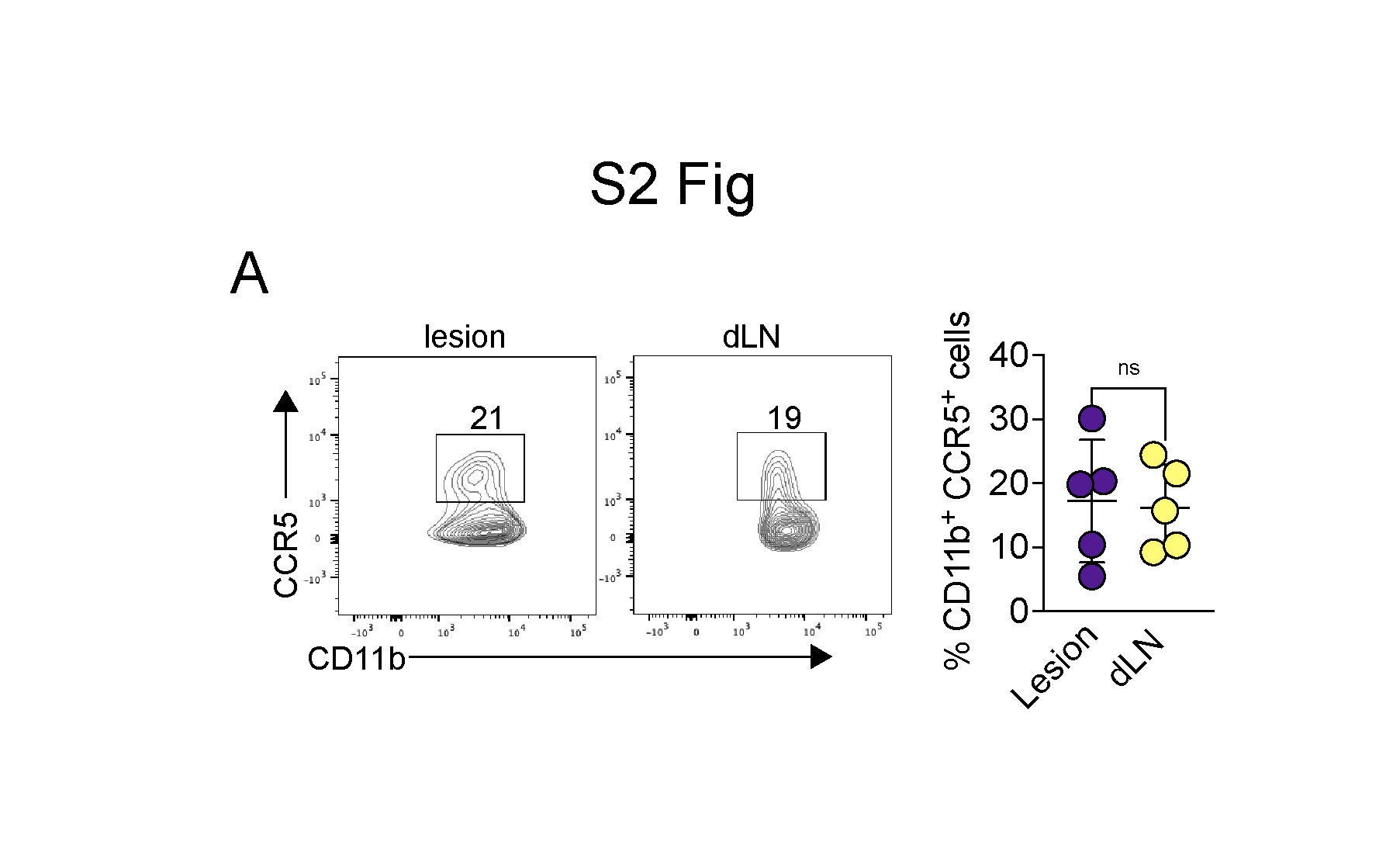


**S4 Fig. CCR5 expression by CD11b^+^ cells in leishmanial lesions.** Representative dot plots and graph bars of CD11b^+^ cells expressing CCR5 in the lesion and dLN of *L. braziliensis*-infected Rag1^-/-^ reconstituted with CD8^+^ T cells at 5 weeks post-infection. Statistical significance was determined using two-tailed unpaired Student's t-test.
